# Trends in Upper Gastrointestinal Bleeding in Children: The Impact of *Helicobacter pylori* Infection and Non-Steroidal Anti-Inflammatory Drug Use

**DOI:** 10.3390/antibiotics13080752

**Published:** 2024-08-10

**Authors:** Felicia Galos, Mara Ioana Ionescu, Mihai Daniel Luca Mirea, Anca Andreea Boboc, Andreea Ioan, Catalin Boboc

**Affiliations:** 1 Department of Pediatrics, Carol Davila University of Medicine and Pharmacy, 020021 Bucharest, Romania; felicia.galos@umfcd.ro (F.G.);; 2 Department of Pediatrics, Marie Curie Emergency Children’s Hospital, 041451 Bucharest, Romania; 3 Department of Functional Sciences, Division of Physiology II—Neuroscience, Carol Davila University of Medicine and Pharmacy, 020021 Bucharest, Romania

**Keywords:** upper gastrointestinal bleeding, *Helicobacter pylori* infection, non-steroidal anti-inflammatory drugs, esophagogastroduodenoscopy, COVID-19 pandemic, children, hematemesis, melena, hematochezia

## Abstract

Upper gastrointestinal bleeding (UGIB) is a significant concern in children, contributing to 6–20% of cases in pediatric intensive care units. This study evaluates the roles of *Helicobacter pylori* (*H. pylori*) infection and non-steroidal anti-inflammatory drug (NSAID) usage in the etiology of UGIB in children, with a particular focus on trends observed during the COVID-19 pandemic. We conducted a retrospective analysis of 103 pediatric patients who underwent esophagogastroduodenoscopy (EGD) for UGIB between January 2015 and December 2023. Of these, 88 patients were included in the final analysis, where the source of bleeding was successfully identified. Hematemesis was the most common presentation, and the source of bleeding was identified in 85.43% of cases. The prevalence of *H. pylori* infection remained stable across the pre-pandemic (39.7%) and post-pandemic (36.7%) periods. However, NSAID usage increased nearly threefold during the pandemic, with 36.7% of post-pandemic UGIB cases associated with NSAID use, compared to 12.1% pre-pandemic. These findings underscore the significant roles of *H. pylori* and NSAID use in pediatric UGIB, with a notable increase in NSAID-related cases during the pandemic.

## 1. Introduction

Gastrointestinal bleeding during infancy and childhood is a prevalent condition, accounting for 6–20% of the pediatric population in pediatric intensive care units. Despite its often-dramatic clinical presentation, the mortality rate associated with gastrointestinal bleeding in children remains relatively low, contrasting sharply with the considerable mortality observed in elderly patients [1].

Upper gastrointestinal bleeding (UGIB) encompasses bleeding originating from the gastrointestinal tract proximal to the ligament of Treitz. It typically manifests as hematemesis (vomiting of blood), melena (black, tarry stools), or hematochezia (passage of fresh blood per rectum). UGIB in children presents unique diagnostic and therapeutic challenges, making it a focal point of pediatric gastroenterology. Among the numerous etiological factors, *Helicobacter pylori* (*H. pylori*) infection and non-steroidal anti-inflammatory drug (NSAID) usage are particularly notable. These factors are common in children and are well-established risk factors for gastrointestinal mucosal injuries [2,3].

*H. pylori* infection is a well-documented etiological factor for various gastrointestinal disorders, including peptic ulcer disease, gastritis, and gastric malignancies, with a concerning increase in bacterial antibiotic resistance [4]. In pediatric patients, *H. pylori* is associated with chronic gastritis and can lead to mucosal damage severe enough to cause UGIB [5,6]. Despite advances in the understanding and treatment of *H. pylori* infection, it remains prevalent in many regions, necessitating ongoing investigation into its role in pediatric UGIB [7].

Non-steroidal anti-inflammatory drugs (NSAIDs) are commonly prescribed to children for their analgesic and anti-inflammatory effects [8]. However, their gastrointestinal side effects, including mucosal erosion, ulceration, and subsequent bleeding, are well-documented. The indiscriminate use of NSAIDs, particularly without adequate gastroprotection, poses a significant risk of UGIB in children. This risk has been further accentuated during the COVID-19 pandemic, where changes in healthcare access, self-medication practices, and increased stress levels may have influenced NSAID consumption patterns [9].

The etiology of UGIB and the proportionate contribution of each cause vary significantly across different geographical regions. This variation is influenced by several factors, including the prevalence of *H. pylori*, the incidence of viral hepatitis, and the age demographics of populations, which in turn affect the burden of non-communicable diseases and their associated morbidities. Over time, changes in the prevalence of *H. pylori* infection, demographic shifts, and the evolving burden of disease in different regions have impacted the types of endoscopic lesions identified in patients undergoing esophagogastroduodenoscopy (EGD) for dyspepsia [10,11].

To provide a more comprehensive analysis of the etiological factors contributing to UGIB in pediatric patients, it is important to consider a range of conditions beyond NSAID usage and *H. pylori* infection, particularly in relation to the patient’s age group. For instance, gastroesophageal reflux (GER) is a significant contributor to UGIB, especially in infants and younger children, where the immature lower esophageal sphincter increases the risk of acid reflux and subsequent mucosal damage [12]. Zollinger–Ellison syndrome, although rare, should be considered in older children and adolescents presenting with recurrent UGIB, as it involves gastrin-secreting tumors leading to excessive gastric acid production [13]. Crohn’s disease is another important factor, particularly in school-aged children and adolescents, where inflammation and ulceration of the gastrointestinal tract can lead to significant bleeding [14]. Additionally, esophageal varices, often associated with chronic liver disease or portal hypertension, are critical to consider in patients of any age presenting with severe UGIB [15].

The COVID-19 pandemic has brought unprecedented changes to healthcare systems globally, influencing patient behaviors and treatment protocols. These changes provide a unique opportunity to investigate the impact of external factors on the etiology and incidence of UGIB in pediatric patients. Understanding these trends is crucial for developing strategies to mitigate UGIB risk and improve patient outcomes during and beyond the pandemic period.

This study aims to evaluate the roles of *H. pylori* infection and NSAID usage in the etiology of UGIB in children over nine years, with a specific focus on the temporal trends observed during the COVID-19 pandemic. By retrospectively analyzing data from pediatric patients who underwent EGD for UGIB, we seek to elucidate the prevalence and impact of these etiological factors. The findings from this study will enhance clinical management strategies and pinpoint specific areas for targeted interventions, ultimately aiming to decrease the incidence and severity of UGIB in pediatric populations.

## 2. Results

### 2.1. Patients’ Characteristics

A total of 103 children, aged between 2 months and 18 years, who underwent EGD over nine years, were evaluated. The source of bleeding was identified in 85.4% (88/103) of the patients, with normal endoscopic findings found in 14.6% (15/103) of the patients. Therefore, only 88 patients were included in the final study.

The median age was 10.98 ± 5.68 years, with a gender distribution of 42.1% males (37/88) and 57.9% females (51/88). Regarding residency, 47.6% (42/88) of the patients were from urban areas, while 52.4% (46/88) were from rural areas. Hematemesis was the most common presentation, observed in 85.2% of the cases (75/88), followed by melena in 25% (22/88) and hematochezia in 3.4% (3/88).

The average interval between the onset of bleeding and the performance of EGD was 2 days. The mean duration of hospitalization for these patients was 8 ± 4.9 days, indicating the typical time required for diagnosis, treatment, and stabilization. The mean hemoglobin level among the patients was 10.5 ± 3.2 g/dL. This value provides insight into the baseline hematological status of the patients upon admission and reflects the severity of blood loss experienced during the UGIB episode.

### 2.2. Causes of UGIB and Endoscopic Findings

**Figure 1:**
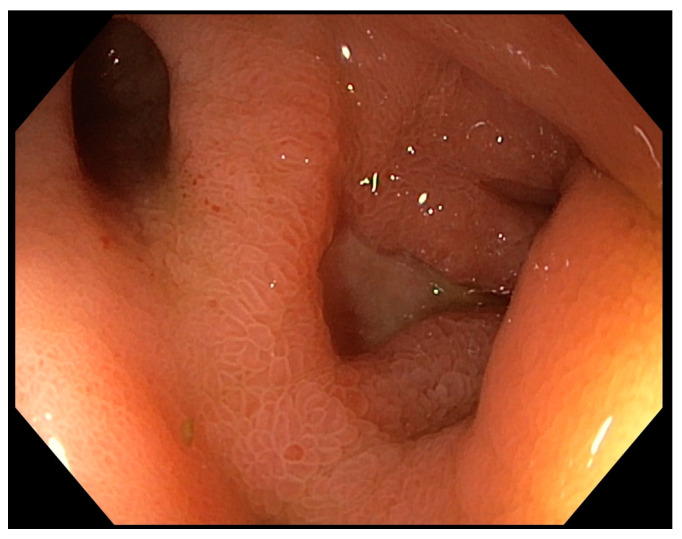
Endoscopic image of the gastric mucosa from a 17-year-old male patient included in this cohort. This image shows a gastric ulcer located in the prepyloric region on the right and a rare finding of a pediatric gastric diverticulum on the left. The detailed visualization of the mucosal texture and vascular patterns aids in the diagnosis and management of these gastrointestinal abnormalities. Image taken from our personal collection.

The distribution of bleeding sources included esophageal causes in 36.4% (32/88), gastric causes in 54.5% (48/88), and duodenal causes in 9.1% (8/88), as presented in Table 1. Specifically, esophageal variceal bleeding was noted in 12.5% of cases (4/88), while non-variceal causes accounted for 87.5% (28/88). Esophagitis and Mallory–Weiss syndrome were observed in 18.2% (16/88) and 5.7% (5/88) of cases, respectively. Gastric causes included erosive gastritis in 28.4% of cases (29/88), nodular gastritis in 21.6% (22/88), and gastric ulcers in 4.5% (4/88, Figure 1). Duodenal findings comprised duodenal ulcers in 6.8% (7/88) and duodenitis in 2.3% (3/88) of patients.

These detailed observations provide a comprehensive overview of the clinical and endoscopic characteristics of pediatric UGIB in this cohort.

To provide a more detailed analysis of the etiology and clinical presentation of UGIB in pediatric patients, we stratified our study population into specific age groups: infants aged 2 months to 1 year, infants aged 1–2 years, and children older than 2 years. We didn’t include neonates since the youngest patient included was 2 months old. The etiology of bleeding in these patients is illustrated in Table 2.

### 2.3. Drug Usage in Patients with UGIB

A history of drug usage was identified in 18 patients (20.4%). Among these patients, 55.6% had used ibuprofen, and 44.4% had used acetaminophen. Additionally, 5.7% (5/18) had a history of antibiotic use, with three patients having used them in combination with NSAIDs and two using them alone. A small proportion, 3.4% (3/18), had recently used corticosteroids.

The endoscopic findings for patients with a history of medication use are detailed in Table 3.

### 2.4. H. pylori Infection Detection

*H. pylori* was detected in 37.5% of the patients. The mean age of patients with *H. pylori* infection was 15 ± 2.8 years, compared to 9.1 ± 6 years in those who tested negative for the infection.

In this study, the most frequent lesion identified by endoscopy was macroscopic antral nodular gastritis, observed in 23 patients. Detailed endoscopic findings in *H. pylori*-positive patients are presented in Table 4.

### 2.5. Pre- and Post-Pandemic Effects on UGIB Patients

When dividing the cohort into pre-pandemic and post-pandemic periods, 65.9% (58/88) of the patients were from the pre-pandemic period, while 34.1% (30/88) were from the pandemic and post-pandemic periods.

Statistical analysis revealed no significant difference in mean age between these periods (*p* = 0.8). The mean age of patients before the pandemic was 10.91 ± 5.56 years, with a gender distribution of 63.8% males (37/58) and 36.2% females (21/58). During and after the pandemic, the mean age was 11.2 ± 6.01 years, with 53.3% males (16/30) and 46.7% females (14/30).

Analysis of residency did not reveal any significant difference in the proportion of patients from urban versus rural areas between the pre-pandemic (48.3% urban, 51.7% rural) and post-pandemic (50% urban, 50% rural) periods (*p* = 0.9).

A notable finding was a nearly three-fold increase in NSAID consumption. Before the pandemic, 12.1% of patients with upper gastrointestinal bleeding had a history of NSAID use, compared to 36.7% during and after the pandemic, which was statistically significant (*p* = 0.01).

The prevalence of *H. pylori* infection remained relatively constant, with 39.7% positivity in the pre-pandemic period and 36.7% in the post-pandemic period (*p* = 0.8).

The average duration from the onset of bleeding to the performance of EGD was 2 days in the pre-pandemic period, decreasing to one day during and after the pandemic.

The mean hemoglobin level decreased from 11.28 ± 3.09 g/dL before the pandemic to 8.67 ± 2.95 g/dL during and after the pandemic (*p* < 0.001).

Additionally, the average duration of hospitalization increased slightly from 5 days in the pre-pandemic period to 6 days during and after the pandemic.

These findings highlight significant changes in clinical presentations, management practices, and outcomes of pediatric UGIB associated with the COVID-19 pandemic.

## 3. Discussion

The present study provides a comprehensive analysis of the etiological factors and clinical outcomes of UGIB in a pediatric population over nine years, with particular attention to the impact of the COVID-19 pandemic. Several notable findings emerged from this investigation, which warrants further discussion.

Our study did not utilize a scoring system for determining the necessity of EGD due to the absence of a validated pediatric system at that time. However, Thomson et al. had previously validated a clinical scoring system in 2015, which included a comprehensive analysis of clinical variables to predict the need for endoscopic intervention [16]. Significant risk factors identified included preexisting conditions, melena, large hematemesis, tachycardia, hemoglobin drop, and the need for fluid bolus or blood products. These variables were weighted and combined to create a predictive scoring system. More recently, Zheng et al. proposed a pediatric scoring system based on clinical and biochemical parameters, derived from an analysis of 224 acute UGIB cases in children with a mean age of 7 years [17]. Despite the intriguing potential of these scoring systems, they are not universally applicable, particularly to infants, due to the limited inclusion of infants in these studies [18].

An Italian multicenter study conducted in 2022 further advanced this field by analyzing records of infants aged between 29 days and 24 months admitted for hematemesis [18]. The study aimed to develop a scoring system based on anamnestic and clinical parameters, along with laboratory and instrumental findings, to identify infants requiring timely EGD. Secondary objectives included assessing the prevalence of recognized causes of hematemesis in infants and evaluating diagnostic and therapeutic approaches [18]. The use of various scoring systems as screening tools can help stratify children with UGIB and determine the need for endoscopic interventions [19].

In our study, endoscopy was primarily performed to identify the cause of bleeding, with therapeutic endoscopy being relatively uncommon (20.4%). The male-to-female ratio was 1.4:1, which aligns with other studies on UGIB in children [20,21]. We were able to identify the source of bleeding in 85.4% of cases, while 14.6% of patients had normal endoscopic findings.

The distribution of patients between the pre-pandemic and pandemic/post-pandemic periods revealed no significant difference in mean age, gender distribution, or residency. This suggests that the demographic characteristics of pediatric patients presenting with UGIB remained stable despite the external pressures and changes brought about by the pandemic.

However, one of the most striking findings was the important increase in NSAID usage during the pandemic period. Before the pandemic, only 12.1% of patients with UGIB had a history of NSAID use. This figure rose to 36.7% during and after the pandemic, indicating a nearly three-fold increase, with a *p*-value of 0.01. This surge in NSAID consumption could be attributed to several factors, including increased over-the-counter use of these medications during the pandemic due to limited access to healthcare services, and heightened self-medication practices [9]. Our data suggest that the gastrointestinal risks of NSAIDs are not confined to adult populations and that similar caution should be applied when prescribing these medications to children. This study adds to the growing body of literature that calls for more stringent guidelines and preventive strategies to mitigate the risks of NSAID use, particularly in vulnerable pediatric groups.

NSAIDs are some of the most extensively used medications globally for their anti-inflammatory and antipyretic effects, widely utilized in both adults and children. They are frequently used for fever control and are generally considered safe. However, their gastrointestinal complications, including peptic ulcer disease, bleeding, and perforation, are well-documented [22]. Grimaldi-Bensouda et al. reported that 46.9% of children with UGIB had taken NSAIDs at least once before the index date, with an adjusted odds ratio of 8.2, emphasizing the significant gastrointestinal risks associated with NSAIDs [23]. This study confirms the necessity of caution when prescribing NSAIDs to children, advocating for alternative therapies, when possible, to avoid these complications.

In our analysis, we included the use of acetaminophen and antibiotics alongside NSAIDs to provide a comprehensive overview of the potential factors contributing to UGIB in pediatric patients. Although NSAIDs are well-documented as a significant risk factor for gastrointestinal bleeding, the inclusion of acetaminophen and antibiotics was intended to explore any potential associations these commonly used medications might have with UGIB. While our data did not show a strong correlation between acetaminophen or antibiotic usage and UGIB, it is important to consider these findings within the broader context of pediatric medication safety. Acetaminophen, although generally considered safe for pain and fever management, can contribute to gastrointestinal issues when used in combination with other medications or in cases of overdose [24]. Similarly, certain antibiotics such as high doses of β-lactams have been associated with gastrointestinal side effects that could exacerbate existing conditions [25]. Therefore, while the primary focus of our study remains on NSAIDs, we chose to retain acetaminophen and antibiotics in our analysis to underscore the importance of considering all potential contributors to UGIB, particularly in complex clinical scenarios where multiple medications may be involved. Further research is needed to better understand these interactions and their clinical implications.

Despite the common practice of self-medication, especially during the pandemic, it is crucial to recognize the associated risks. Patients and parents often misunderstand dosages and contraindications, highlighting the critical role of pharmacists in providing accurate information about medication use [26]. Tarciuc et al. found that the pandemic did not significantly alter mothers’ approaches to managing their children’s acute illnesses, suggesting that parents achieved a degree of self-treatment awareness due to the pandemic’s context [9].

The prevalence of *H. pylori* infection remained relatively stable between the pre-pandemic (39.7%) and during and post-pandemic (36.7%) periods, with a *p*-value of 0.8. This stability aligns with findings in adults, where a decrease in *H. pylori*-positive peptic ulcers has been observed, coupled with an increase in NSAID-associated ulcers [27]. However, in children, data on the trend of *H. pylori* prevalence in peptic ulcer disease are limited. A European multicenter pilot study found that ulcers occurred in 8.06% of pediatric cases, with *H. pylori* present in only 27% of these cases [28,29].

Other studies support these findings, such as one where 454 children with *H. pylori*-associated gastritis included 64 with ulcers, reflecting a significant regional variation in ulcer prevalence [29]. This study underscores the importance of continuous monitoring and targeted interventions for managing UGIB in pediatric populations effectively.

Our study demonstrated that both *H. pylori* infection and NSAID usage are significant factors in the etiology of UGIB in children. However, it is important to note that while the prevalence of *H. pylori* infection remained relatively constant across the pre-pandemic, pandemic, and post-pandemic periods, it continues to be a substantial risk factor for UGIB. The most significant finding of our study is the marked increase in NSAID-related UGIB cases during the pandemic and post-pandemic periods. This suggests a shift in the predominant risk factors contributing to UGIB, with NSAID usage becoming increasingly prominent during this time. Consequently, while *H. pylori* remains a key consideration in the management of UGIB, the rising use of NSAIDs, especially during global health crises like the COVID-19 pandemic, highlights the need for targeted interventions to mitigate the associated risks.

To address the relationships between the factors studied and the etiology of UGIB, it is important to clarify that while our findings suggest associations between NSAID usage, *H. pylori* infection, and the incidence of UGIB in pediatric patients, these relationships should not be interpreted as direct cause-and-effect without further investigation. Specifically, the consistent prevalence of *H. pylori* across the study periods indicates its ongoing relevance as a risk factor for UGIB, but the absence of temporal variation during the COVID-19 pandemic suggests that *H. pylori* alone may not fully account for the observed trends in UGIB incidence. On the other hand, the marked increase in NSAID-related UGIB cases during the pandemic highlights a potential causative link, driven by changes in medication practices during this period. However, this study’s design does not allow for definitive conclusions regarding causality, and we recommend that future research employs more rigorous methods, such as prospective studies or randomized controlled trials, to better understand these complex interactions.

Additionally, the average duration from the onset of bleeding to the performance of EGD decreased from 2 days pre-pandemic to 1 day during and post-pandemic. This reduction suggests improved efficiencies in emergency response and diagnostic processes, potentially driven by adaptations necessitated by the pandemic. Quicker diagnostic interventions are crucial for the timely management of UGIB and could positively impact patient outcomes.

The important decrease in mean hemoglobin levels from 11.28 g/dL pre-pandemic to 8.67 g/dL during and post-pandemic indicates more severe anemia in children presenting with UGIB during the pandemic. This severity might reflect delays in seeking care or increased disease severity. Furthermore, the increased duration of hospitalization from 5 days pre-pandemic to 6 days during and post-pandemic supports the notion that patients presented with more severe conditions requiring extended care.

## 4. Materials and Methods

### 4.1. Study Design

We conducted a retrospective observational study at Marie Sklodowska Curie Emergency Children’s Hospital, a tertiary care center in Bucharest, Romania. The study included pediatric patients who underwent EGD for UGIB between January 2015 and December 2023. The pre-pandemic period was defined as the time before February 2020, when the first case of COVID-19 was reported. The pandemic and post-pandemic period was defined as the time from February 2020 onwards.

### 4.2. Patient Inclusion Criteria

Patients were included if they presented with symptoms of UGIB such as hematemesis, melena, or both and underwent EGD. Moreover, they were included if during the EGD a clear source of bleeding was identified.

### 4.3. Data collection and patient information

Data were systematically extracted from medical charts for all enrolled patients. The collected data included demographic and clinical information such as age at the onset of UGIB, gender, and residency (urban or rural area). Information on the history of drug intake, clinical presentation, endoscopic findings, and the presence of *H. pylori* in gastric biopsy samples was also recorded. Laboratory data such as hemoglobin levels from the patients were collected. Furthermore, information on diagnostic investigations, prescribed therapy, hospital admission status and length of hospital stay, presumed or confirmed diagnosis, and outcome at follow-up was compiled. Data regarding the patient’s history of *H. pylori* infection and previous therapies were also obtained.

### 4.4. Diagnostic Procedures

All patients underwent EGD performed by experienced pediatric gastroenterologists. Gastric biopsy samples were obtained during the procedure to test for *H. pylori* infection using standard histopathological techniques, except in patients with esophageal variceal bleeding, where the intervention was therapeutic. This procedure was conducted on patients who had fasted for a minimum of 10 h, except in emergencies, and was performed under either general anesthesia or conscious sedation. Throughout the procedure, vital signs were continuously monitored. Written informed consent was obtained from the parent or guardian of each child.

### 4.5. Histology

**Figure 2:**
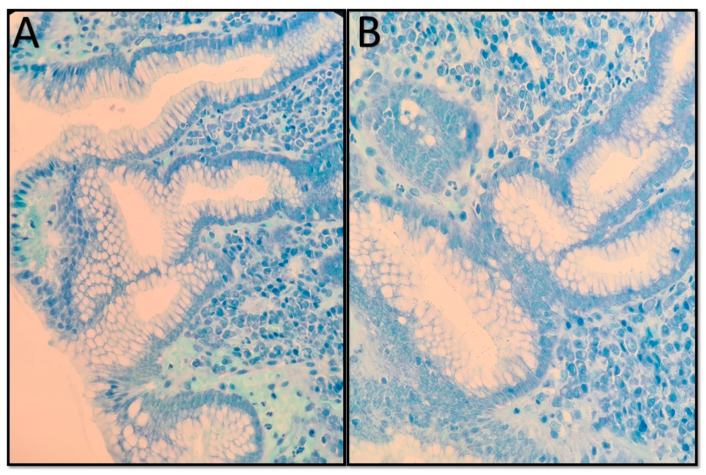
Microscopic image showing *Helicobacter pylori* bacilli in a gastric biopsy from a pediatric patient included in this cohort, using Giemsa stain at 400x magnification. The bacilli appear as short, curved, or spiral-shaped organisms adhering to the gastric epithelial surface. Panel (**A**) shows a mild infiltration of inflammatory cells with presence of *H. pylori* bacilli on the surface of the epithelial cells. Panel (**B**) exhibits a more severe inflammatory response with denser infiltration of inflammatory cells, also showing *H. pylori* bacilli. Images taken from our personal collection.

During the endoscopic procedure, two biopsy specimens each were taken from the duodenum, antrum, corpus, and esophagus and fixed in 10% formaldehyde. The gastric mucosa samples underwent standard dehydration and paraffin embedding processes. Histological examination was performed using hematoxylin and eosin (H&E) and Giemsa stains. H&E stain was primarily used to evaluate inflammatory cells and detect *H. pylori* [12]. When H&E staining failed to identify the bacterium, Giemsa stain was used as a supplementary method. *H. pylori* appeared in histological sections as short, curved, or spiral bacilli on the epithelial surface or within the mucus layer (Figure 2).

### 4.6. Data Analysis

The data were analyzed using GraphPad Prism 9.3.0 software (GraphPad Software Inc., San Diego, CA, USA). We have used descriptive statistics as a statistical method to summarize and describe the essential characteristics of the dataset. Specifically, these statistics include measures such as mean, median, standard deviation, and proportions, which provide a clear overview of patient demographics, clinical presentations, and other relevant variables related to UGIB in the study population. Descriptive statistics were employed to present the distribution of these variables in a straightforward manner, allowing us to effectively communicate the central tendencies and variability within the data. This approach was crucial in identifying patterns and trends, such as the frequency of NSAID usage and the prevalence of *H. pylori* infection, and served as the foundation for further inferential statistical analysis. The differences in the prevalence of *H. pylori* infection and NSAID usage between pre-pandemic and post-pandemic periods were assessed using Fisher’s Exact for low expected frequencies. A *p*-value of less than 0.05 was considered statistically significant.

### 4.7. Ethical Considerations

The study protocol was approved by the Institutional Review Board of Marie Sklodowska Curie Emergency Children’s Hospital. Given the retrospective nature of the study, informed consent was waived. All patient data were anonymized to ensure confidentiality and adherence to the ethical standards outlined in the Declaration of Helsinki.

## 5. Conclusions

In conclusion, this study highlights the significant changes in NSAID usage and clinical severity of UGIB in pediatric patients during the COVID-19 pandemic. These findings underscore the importance of cautious NSAID use and the need for timely medical intervention to manage UGIB effectively. Our study emphasizes that *H. pylori* remains a significant and persistent risk factor for gastrointestinal bleeding in pediatric populations. Although the prevalence of *H. pylori* infection did not exhibit significant changes between the pre-pandemic, pandemic, and post-pandemic periods, its constant presence underscores its ongoing relevance as a contributing factor to UGIB. The stability in *H. pylori* prevalence highlights that while it continues to be a critical consideration in managing UGIB, the notable increase in UGIB cases during the pandemic was more strongly associated with the rise in NSAID usage rather than changes in *H. pylori* infection rates. Thus, our findings suggest that, although *H. pylori* remains an important etiological factor, the pandemic’s impact on UGIB incidence was predominantly driven by increased NSAID consumption. This distinction is crucial for guiding future clinical management strategies, where both *H. pylori* infection and NSAID use must be carefully monitored and managed to reduce the risk of UGIB in pediatric patients.

## Figures and Tables

**Table 1:** Causes of upper gastrointestinal bleeding based on the anatomic location.

Source of Bleeding		Percentage (Absolute Value)
Esophageal-related causes (32/88)	Variceal bleeding	12.5% (11/88)
	Mallory–Weiss syndrome	5.7% (5/88)
	Esophagitis	18.2% (16/88)
Stomach-related causes (48/88)	Erosive gastritis	28.4% (25/88)
	Nodular gastritis	21.6% (19/88)
	Gastric ulcer	4.5% (4/88)
Duodenal-related causes (8/88)	Duodenitis	2.3% (2/88)
	Duodenal ulcer	6.8% (6/88)

**Table 2:** Causes of upper gastrointestinal bleeding in pediatric patients stratified by age.

Patient Category	Number of Patients	Causes of UGIB
Infants (2 months to 1 year)	9.1% (8/88)	Variceal bleeding—2/88 Mallory–Weiss syndrome—2/88 Esophagitis—1/88 Gastritis—2/88 Gastric ulcer—1/88
Children (1 year to 2 years)	4.5% (4/88)	Esophagitis—2/88 Duodenal ulcer—2/88
Older children (more than 2 years)	86.4% (76/88)	Variceal bleeding—9/88 Mallory-Weiss syndrome—3/88 Esophagitis—13/88 Gastritis—42/88 Gastric ulcer—3/88 Duodenitis—2/88 Duodenal ulcer—4/88

**Table 3:** Endoscopic findings depending on medication use.

Type of Used Drug	Endoscopic Findings	Number of Patients
Ibuprofen	Mallory–Weiss syndrome	5.6% (1/18)
	Esophagitis	16.7% (3/18)
	Erosive gastritis	11.1% (2/18)
	Nodular gastritis	11.1% (2/18)
Acetaminophen	Esophagitis	11.1% (2/18)
	Erosive gastritis	27.8% (5/18)
	Duodenal ulcer	16.7% (3/18)
Antibiotics	Esophagitis	5.6% (1/18)
	Erosive gastritis	11.1% (2/18)
	Duodenal ulcer	11.1% (2/18)
Corticosteroids	Erosive gastritis	5.6% (1/18)
	Duodenal ulcer	11.1% (2/18)

**Table 4:** Endoscopic Findings in *H. pylori*-positive patients.

Endoscopic Findings	Number of Patients
Esophagitis	18.2% (6/33)
Erosive gastritis	3.0% (1/33)
Nodular gastritis	69.7% (23/33)
Gastric ulcer	3.0% (1/33)
Duodenal ulcer	6.1% (2/33)

## Data Availability

The original contributions presented in the study are included in the article, further inquiries can be directed to the corresponding author/s.
